# Misaligned and Polarity-Reversed Faces Determine Face-specific Capacity Limits

**DOI:** 10.3389/fpsyg.2016.01470

**Published:** 2016-09-27

**Authors:** Volker Thoma, Neil Ward, Jan W. de Fockert

**Affiliations:** ^1^School of Psychology, University of East LondonLondon, UK; ^2^Department of Psychology, Goldsmiths, University of LondonLondon, UK

**Keywords:** attention, face perception, perceptual load, capacity limits, flanker paradigm, holistic processing, polarity reversal

## Abstract

Previous research using flanker paradigms suggests that peripheral distracter faces are automatically processed when participants have to classify a single central familiar target face. These distracter interference effects disappear when the central task contains additional anonymous (non-target) faces that load the search for the face target, but not when the central task contains additional non-face stimuli, suggesting there are face-specific capacity limits in visual processing. Here we tested whether manipulating the format of non-target faces in the search task affected face-specific capacity limits. Experiment 1 replicated earlier findings that a distracter face is processed even in high load conditions when participants looked for a target name of a famous person among additional names (non-targets) in a central search array. Two further experiments show that when targets and non-targets were faces (instead of names), however, distracter interference was eliminated under high load—adding non-target faces to the search array exhausted processing capacity for peripheral faces. The novel finding was that replacing non-target faces with images that consisted of two horizontally misaligned face-parts reduced distracter processing. Similar results were found when the polarity of a non-target face image was reversed. These results indicate that face-specific capacity limits are not determined by the configural properties of face processing, but by face parts.

## Introduction

In modern daily life, people see many human faces, and increasingly this happens by looking at images (e.g., in photographs and social media). Despite sharing the same basic parts (eyes, nose, mouth), recognition of individual faces appears to be fast and almost effortless in normal circumstances. One reason for the apparent ease of face recognition is the ability of the visual system to recognize a face as a whole, rather than process its individual features in a piece-meal fashion (Young et al., 1987; Tanaka and Farah, 1993; Laguesse and Rossion, 2013). However, there is recent evidence that only a limited number of faces can be recognized in parallel (Thoma and Lavie, 2013) indicating that face recognition has a limited capacity. The current study investigates whether face-specific capacity limits are associated with mental representations that rely on part-processing or processing of the whole face.

For some time, experimental evidence has suggested that face recognition is based on “automatic” processes that are deemed to be fast (Young et al., 1986), difficult to suppress intentionally (Wojciulik et al., 1998), and require only minimal attentional resources (Schneider and Chien, 2003, see Palermo and Rhodes, 2007, for a review). Human faces are also processed faster than any other visual category, including ape faces (Itier et al., 2011). Accordingly, one would expect face recognition to be relatively unhindered by limits in processing capacity, and only minimally affected if demand for visual attention (and therefore processing capacity) was allocated elsewhere. This was indeed observed in a number of behavioral (Jenkins et al., 2002; Reddy et al., 2004) and neuro-physiological studies (Neumann and Schweinberger, 2008).

One account that explicitly predicts capacity-limits in visual processing is perceptual load theory (PLT; Lavie and Tsal, 1994; Lavie, 1995; Lavie et al., 2004). The theory holds that, in tasks with low perceptual load (e.g., when the search for a visual target is undemanding because non-targets are few or easy to distinguish from the target), spare attentional capacity remains available for processing irrelevant distracters. However, at higher levels of perceptual load an irrelevant distracter is hardly or not at all processed (Lavie, 1995, 2005; Lavie and Cox, 1997) because the main task does not leave any spare capacity. In a typical experimental paradigm using binary categorization, Lavie et al. (2003; Experiment 2) asked subjects to search the center of a computer screen for the name of an object, among one, two, four, or six non-word letter strings, and categorize it as either belonging to the category of fruits or musical instruments, whilst ignoring a distracter image in the periphery. The distracter was either a photograph of the target (congruent condition) or a photograph from the opposite category (incongruent condition). The experiment showed faster response times in the congruent compared to the incongruent condition, indicating that the distracter image was processed, and—as predicted by load theory—this congruency effect was eliminated when the set size of non-targets in the center was increased.

But whereas perceptual load theory seems to adequately account for the fate of processing peripheral letters (Lavie and Cox, 1997) and objects (Lavie et al., 2009), the experimental evidence is different for faces as distracter stimuli. In a target search for letters (Jenkins et al., 2002) or names (Lavie et al., 2003), interference from task-irrelevant faces was not eliminated under high levels of task load. It was thus proposed that the apparent special status of faces may involve “automatic” processing at an early perceptual stage, which would be consistent with the theory that face processing is mediated by a specialized visual module in the brain (Fodor, 1983), triggered automatically in the presence of faces (Kanwisher et al., 1997; Farah et al., 1998). Indeed, there is evidence that recognition of faces is subject to rapid processing in comparison to non-face objects (Young et al., 1986) and that processing appears to be mandatory, meaning that it cannot be prevented at will (Wojciulik et al., 1998; Boutet et al., 2002; Palermo and Rhodes, 2007).

Despite these findings of preserved processing of peripheral faces under attentional load, recent research indicates that there are conditions when the processing of peripheral faces is reduced by capacity limits. Bindemann et al. (2005) showed that when participants categorized centrally shown names of famous people or national flags (as belonging to either the UK or US), famous distracter faces produced response competition effects, but these were eliminated when a face had to be categorized as a central target. A similar finding using priming measures was reported by Bindemann et al. (2007). Thus, it appears that processing of face distracters is capacity-free as long as a central task is not involved with face recognition as well.

To investigate whether these presumed category-specific capacity limits are apparent when the perceptual load of relevant processing is systematically varied, Thoma and Lavie (2013) conducted a series of experiments in which participants searched for the face of a famous politician or pop star and made speeded classification responses. Perceptual load was manipulated through changes in the relevant search set size by adding non-famous faces appearing with the target in the center of the screen. A task-irrelevant face that was the same as the target, or from a different category, was shown in the periphery. As in traditional perceptual load studies, faster and more accurate responses to a target face were observed when the distracter face was the same as the target, rather than from a different category, and this congruency effect was only observed when a single face was presented in the search array. Under high load, when additional non-target faces were added to the search set, the congruency effect was eliminated, indicating a maximum capacity of two to three faces. In a further experiment, Thoma and Lavie replicated the results of Lavie et al. (2003; Experiment 2), which demonstrated that, in a central name search task, response competition effects from incongruent peripheral face images are not affected by increases in perceptual load, removing the possibility that the face-specific perceptual load effects were due to inequity in the load manipulations between the face and name search tasks.

The results of Thoma and Lavie (2013) therefore showed that the processing of face distracters only depends on perceptual load when load manipulation involved face stimuli. Recently, Thoma (2014) confirmed the face-specific aspect of load capacity in similar experiments. Importantly, that study also showed that when the central task was loaded with inverted non-target faces (while searching for an upright famous target face) the congruency effects were still reduced, just as observed with upright non-target faces. This was a surprising finding, as traditionally face recognition research makes a distinction between holistic processing of a whole face and “featural” processing, in which parts of the face are processed separately (Tanaka and Farah, 1993), in a way similar to that observed for processing non-face objects (Maurer et al., 2002). Holistic processing involves rapid classification through integration of facial features—eyes, nose, mouth—which show an established, first-order spatial relationship^1^. Second-order relations, such as the metric distance between facial features, may then be processed to discriminate between faces (sometimes distinguished as “configural” processing, see Richler and Gauthier, 2014). Holistic processing has been originally assumed to occur only when faces are in the upright orientation (Farah et al., 1998), and face recognition can be disrupted by introducing changes in spatial information, for example by presenting a face in an inverted orientation (Nederhouser et al., 2007). Inversion of faces is commonly believed to lead to more part-based processing, whilst having little disruptive effect on processing of the facial features themselves (Searcy and Bartlett, 1996). This so-called face inversion effect (FIE; Yin, 1969) is regularly cited as important evidence that faces have a special status, since it demonstrates that inversion has a greater effect on recognition of a face than on recognition of other objects (but see Richler et al., 2011, for the view that upside-down faces may still be processed “holistically”). Yet, Thoma's (2014) finding that increasing perceptual load with upside-down faces also reduces distracter processing is strong evidence that the observed face-specific capacity limits are not—or not solely—determined by holistic face representations, at least in the sense of so-called first order relations between parts. This leads to the question which other properties of face processing can explain category-specific load effects? One possibility is that the unique range of distinctive spatial frequencies (inherent in images of faces) is responsible for the observed capacity limits. The spatial frequencies present in a face image are the same for upright and upside down faces, but different to other non-face objects or letters (De Valois and De Valois, 1980; Costen et al., 1996), which would account for the findings of both Lavie et al. (2003) and Thoma (2014). However, previous experiments show that scrambled versions (which also retain the spatial frequencies of the original face) of distracter (peripheral) faces did not reduce congruency effects compared to the presence of an intact anonymous face. Thoma and Lavie (2013) also ruled out that spatial frequency determined face capacity limits (see Thoma, 2014, and Discussion Section for details).

The observation that there are no capacity effects from non-target faces with scrambled spatial frequency components, while at the same time face capacity effects persist with inverted faces therefore suggests that face recognition limits are determined by the processing of specific face parts or local features rather than holistic face representations. Indeed, this concurs with recent evidence that face perception relies more on local facial characteristics than previously thought (Gaspar et al., 2008; Schwaninger et al., 2009; Gold et al., 2012). However, inversion of a face may affect face processing in a variety of ways: it may impede the computation of distances between parts such as the nose and eyes (which is thought to underlie face identification (Kemp et al., 1990; Bruce et al., 1991), or it may affect the way information about face parts is sampled (Gaspar et al., 2008; Gold et al., 2012). Recently, Hayward et al. (2016) showed that holistic processing captures both configuration-based and component-based information. Therefore, Thoma's (2014) findings that even inverted non-target faces eliminate target-distracter congruency effects, just as upright faces do, could be explained by face processing capacity relying on processing of parts rather than the first-order relations between them.

Another transformation that impairs the recognition of a face, whilst preserving identifiable features, is based on the Composite Face Effect (CFE; Taubert and Alais, 2009; Laguesse and Rossion, 2013). This is derived from the Composite Face Illusion (CFI) in which the top and bottom halves of two different individual faces are combined into a single composite, or chimeric image, making it more difficult to name the target top half of a familiar face, compared to when it is presented shifted sideways along the horizontal axis (Young et al., 1987). Even if two identical top halves are shown side by side, they are not perceived as from the same face if combined with bottom halves from two different individuals. This striking visual illusion (see Rossion, 2013) shows that aligned half faces cannot be perceived as independent from each other, and is strong evidence that faces are normally perceived as integrated wholes rather than perceived as a collection of features. This integration of the facial features into a Gestalt (a global picture) is reminiscent of the idea of “configural” (Sergent, 1984; Young et al., 1987) or “holistic” (Tanaka and Farah, 1993; Farah et al., 1998) processing—similar to the arguments regarding the inversion effect.

Several mechanisms may underlie the CFE. The misalignment between the two half faces increases the relative distance between the parts in the two halves, which may make individuation of each face easier (Diamond and Carey, 1986; Mondloch et al., 2003). If this were the case, then one would expect a linear relationship between degree of misalignment and the magnitude of the CFE. However, Taubert and Alais (2009) report that the degree of CFE did not differ between two levels of alignment (25% vs. 50%). More recently, Laguesse and Rossion (2013) have shown that holistic processing is reduced when the half-faces are displaced horizontally by as little as 8.3% of the width of the face. Thus, there seems to be a qualitative breakdown of the perceptual whole—i.e., the first-order configuration of the features (Maurer et al., 2002; McKone et al., 2007)—when face halves are even slightly misaligned. This would then lead to more featural processing, similar to the assumed effect of face inversion. We therefore predict that using misaligned faces as non-targets in a visual search set will result in similar effects on target-target congruency as was observed when inverted faces were used (Thoma, 2014).

A third type of image manipulation that has repeatedly been shown to disrupt the processing of faces is to create a negative of the original photo image (Galper, 1970; Phillips, 1972; Johnston et al., 1992). Reversing the contrast polarities of an image (also termed polarity reversion or negation) makes black areas white, light gray areas dark gray, and so forth. Like face inversion, the disruptive effects of polar reversal on face recognition have been observed consistently across a number of experimental paradigms, (Vuong and Tarr, 2004; Nederhouser et al., 2007) although there are differences in interpreting the mode of disruption. Some researchers have proposed that polarity reversal alters shading cues in a face, which impairs interpretation of its three-dimensional properties (Kemp et al., 1990; Johnston et al., 1992). It has also been suggested that polarity reversal disrupts the perception of second-order relations, such as the distance between facial features, which are widely accepted to play an important role in the perceptual representation of faces (Diamond and Carey, 1986; Hole et al., 1999; White, 2001). However, more recent evidence supports the hypothesis that the disruptive effects of polarized faces is driven by the resulting changes in surface pigmentation; i.e., their variation in reflectance (Bruce and Langton, 1994; Vuong and Tarr, 2004; Nederhouser et al., 2007). Notably, Liu et al. (2000) found that recognition was poor for faces missing surface pigmentation (but with intact 3D information). In other studies, employing faces with a similar pigmentation pattern but differing shape (Russell et al., 2006) or non-pigmented faces (Bruce and Langton, 1994), there was little or no effect of polarity reversal on face matching (but see Gilad et al., 2009, that polarity-reversal effects may be limited to some face parts). Whatever the reasons, neurophysiological evidence suggests different mechanisms between inversion and polarity reversal: Itier (Itier and Taylor, 2002) reported that electro-encephalogram (EEG) recordings showed different neural sources of early (P1) effects resulting from inversion compared to polarity reversal effects (see also Itier et al., 2006, for similar results with MEG). The research literature therefore suggests that CFE and polarity reversal, like face inversion, specifically affect face recognition, but not—or only to a limited degree—recognition of non-face objects (Subramaniam and Biederman, 1997; Nederhouser et al., 2007).

We tested two predictions. If processing of misaligned half faces (presumed to be non-holistic in the sense of changed second-order relationships between parts) and/or polarity reversed faces (either affecting second-order relationships or face-part recognition itself) relies on the same processing capacity as does the processing of intact faces, then we expect that the presence of misaligned and polarity reversed faces respectively will reduce the processing of peripheral distracter faces (like upright and inverted faces do; Thoma, 2014). If, however, the nature of processing misaligned and polarity reversed faces means that they do not share processing resources with intact faces, then the misaligned and polarity reversed faces will impose fewer capacity demands, and peripheral distracter faces should receive processing (similar to the low load conditions in Thoma and Lavie, 2013). We predicted that if face-specific capacity limits are determined by face parts or features (Gold et al., 2012) rather than configural properties (Maurer et al., 2002; Laguesse and Rossion, 2013) then we would expect that only the misaligned face manipulation but not contrast reversal will load a face-specific capacity.

The current investigation includes three experiments. Experiment 1 aimed to confirm that interference from distracter faces occurs irrespective of task load for non-face targets (as first reported by Lavie et al., 2003) and two further experiments examine the effects of disrupting configural face processing on face-specific load capacity using the CFE (Young et al., 1987) and polarity reversal (Galper, 1970).

## Experiment 1

**Figure 1:**
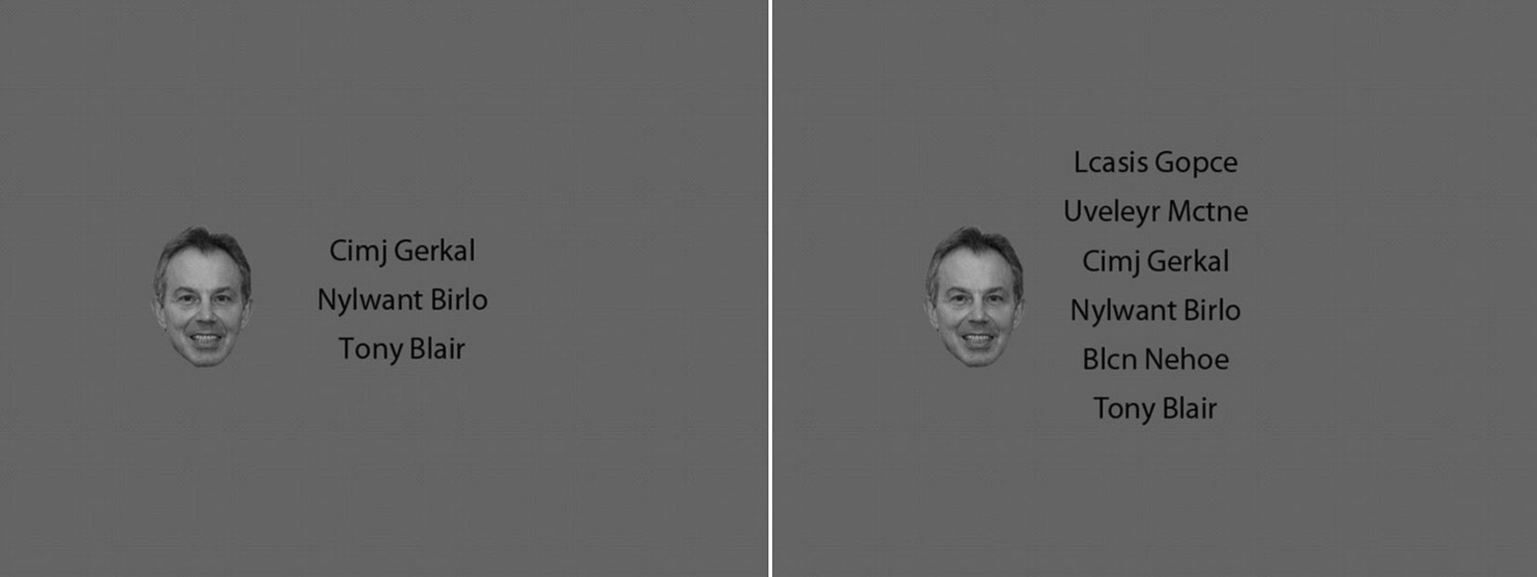
**Examples of displays in Experiment 1**. Shown is a congruent display with a relevant set size of three items **(left panel)** or six items (**right panel**; see caption of Figure 3 for copyright information on the face images).

Experiment 1 employed a visual search and binary classification task similar to that first used by Lavie et al. (2003) and which was replicated in Thoma and Lavie (2013; Experiment 2). In each trial, participants classified the name of a famous male politician or film star in displays of either low (target name plus two non-target name-like letter strings) or high (target name plus five non-target name-like letter strings) perceptual load. In all conditions, the face of a famous politician or film star was presented in the periphery (see Figure 1). The key measure of interest was the effect of the congruency between the target name and the distracter face on response latencies and accuracy, as a function of perceptual load.

### Materials and methods

#### Participants

Participants were recruited from the student body at the University of East London and all reported normal or corrected-to-normal vision. Potential participants were asked to name eight famous faces from the images used in the experiment, which included four male politicians (David Cameron, Tony Blair, George Bush and Bill Clinton) and four male film stars (Hugh Grant, Robert DeNiro, Daniel Craig, and George Clooney). Sixteen people (mean age 21.3, *SD* = 2.5; 5 males) who could name all eight faces participated without compensation. Written consent was obtained and the study was approved by the Ethics committee of the University of East London.

#### Stimuli and procedure

Participants were placed in front of a 15” CRT monitor at a distance of approximately 60 cm. They were asked to attend to the center of the display and classify a target name as that of a famous politician or a film star through a key press, whilst ignoring a peripheral distracter face. In the low load condition, there were two additional non-target letter strings in the search area. The famous name was displayed in one of six vertical positions (rows), with two of the other (adjacent, or both above or below) rows filled by name-like non-sense letter strings. In the high load condition, the famous name was displayed in one row and all five remaining rows were filled by non-sense letter strings. All non-targets were non-sense letter strings in a first name-last name format, e.g., “Cgerth Jnfedgsa.” The distracter face either matched the target name (congruent condition) or was selected from the faces in the other category (incongruent condition).

The relevant search display was presented in a vertical column in the center of the display. Target and non-target letter stimuli were shown in Arial 12 bold, and the horizontal expanse of the letter strings was between 3.5 cm (3.34 degrees) and 4.9 cm (4.68 degrees). The vertical expanse from the top edge to the bottom edge was 3 cm (2.86 degrees) in the low load condition and 6 cm (5.73 degrees) in the high load condition. Distracter face images were presented in grayscale with a standardized vertical size of 3.4 cm (3.24 degrees) and positioned at the periphery of the screen 4 cm (3.82 degrees) to the left or right of fixation.

E-prime 1.1 was used to run the experiment and counterbalancing was applied regarding the target category (politician vs. films star), identity, and positions of the target (six positions) and distracter (left or right). Participants ran through a practice block of 96 trials followed by 4 experimental blocks of 96 trials each, with conditions randomly intermixed in each block. Displays remained visible for 3 s unless the participant responded sooner. Response times and error rates were analyzed using parametric tests, except when assumptions for normal distribution of data were violated (non-parametric tests were then used, for error rates) or the assumption of sphericity (as happened for RTs, Greenhouse-Geisser corrections were then used).

### Results

Only correct response times (RTs) greater than 150 ms were analyzed; trials with responses faster than 150 ms were excluded (1.5% of trials). A two-way, within-subjects Analysis of Variance (ANOVA) was carried out on correct RTs. There were two levels of load, set size three (low load) and set size six (high load), and two levels of congruence (congruent vs incongruent) for the distracter face relative to the target name.

**Figure 2:**
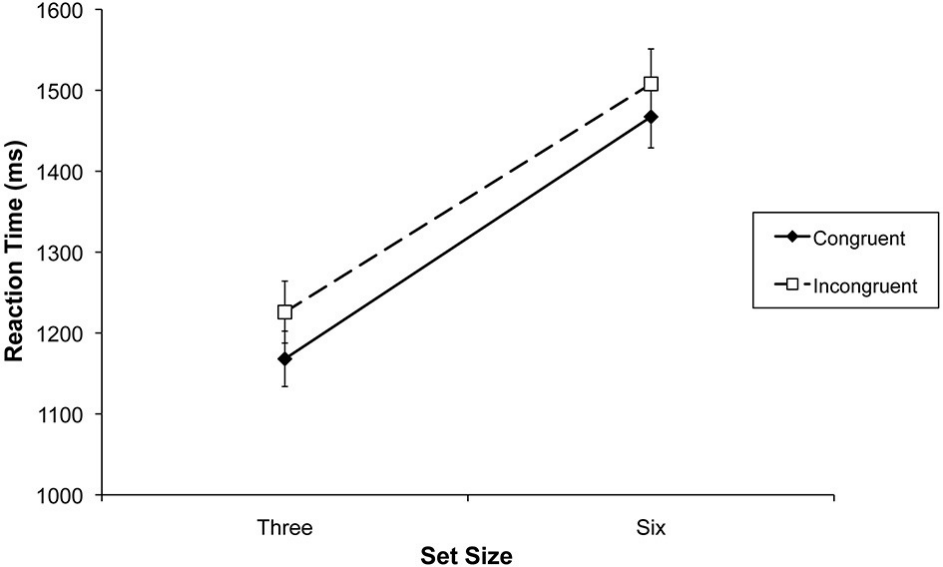
**Mean reaction times in the name classification task of Experiment 1 as a function of set size and congruency**. Error bars represent standard error of the mean.

In the RTs there was a significant main effect of load, *F*_(1, 15)_ = 336.3, *p* < 0.001, partial η^2^ = 0.95. RTs were faster under low load (*M* = 1197, *SD* = 140) compared to high load (*M* = 1488, *SD* = 158). The main effect of congruency was also significant, *F*_(1, 15)_ = 8.42, *p* = 0.011, partial η^2^ = 0.36. RTs (see Figure 2) were faster on congruent trials (*M* = 1318, *SD* = 140) compared to incongruent trials (*M* = 1366, *SD* = 158). Importantly, there was no interaction between load and congruency, *F*_(1, 15)_ = 0.5, *p* = 0.48, indicating that the congruency effect produced by the distracter faces remained unchanged as a function of load.

The congruency effect was significant for set size 3 [*t*_(1, 15)_ = 2.49, *p* = 0.025] and set size 6 [*t*_(1, 15)_ = 2.26, *p* = 0.039]. An analog analysis of the error rates in each condition (overall *M* = 8%, *SD* = 7%) did not reveal any significant main effects or an interaction (all *F*s < 1.19; See Table 1).

**Table 1:** **Mean error rates (in percent) and Standard deviation for conditions in Experiment 1**.

**Error rate**	**Congruent**		**Incongruent**	
	***M***	***SD***	***M***	***SD***
Set size 3	0.08	0.06	0.09	0.05
Set size 6	0.07	0.04	0.09	0.05

The main effect of load in the RT analysis confirmed that load was successfully manipulated. Nonetheless, the congruence effect was unaffected by increasing load with non-face stimuli, suggesting that the processing of distracter faces was independent of the attention required for processing the central non-face stimuli. This result therefore replicates findings with almost identical paradigms in Lavie et al. (2003) and Thoma and Lavie (2013).

## Experiment 2

In Experiment 1, task-irrelevant faces were processed irrespective of the attentional demands of the relevant task, which could suggest (i) that face recognition is capacity free, or (ii) that face processing has capacity limitations, but that it does not compete for resources with processing non-face information (the relevant names in this case). The previous finding that increasing the attentional demands of the relevant task by adding face stimuli to the relevant set does modulate the processing of peripheral distracter faces (Thoma and Lavie, 2013; Thoma, 2014), suggests that face processing is subject to capacity limitations, but that these are face-specific. The question remains which aspects of face processing drive the face-specific capacity limitation. Since processing of inverted faces was found to consume capacity (Thoma, 2014), holistic face processing appears not to be a necessary condition to exhaust face-specific capacity. Experiment 2 was designed to further test this assertion, by presenting a to-be-recognized target face together with either intact or chimeric non-target faces. In line with Thoma and Lavie (2013), we predicted that intact non-target faces would eliminate the congruency effect produced by peripheral distracter faces. The key effect of interest was the congruency effect for displays containing misaligned non-target faces. If such faces are able to consume capacity despite not being processed as a face-like configural whole, we predicted a reduction in the distracter congruency effect, similar to the previous finding using inverted faces (Thoma, 2014). Such a finding would suggest that face-capacity limits are determined by non-configural representations of faces.

### Materials and methods

#### Participants

Twenty (nine male) were recruited on a voluntary, unpaid basis, among psychology students at the University of East London. The mean age of participants was 23.15 (*SD* = 4.35) with ages ranging from 18 to 34. All reported normal or corrected-to normal vision. Participants read a document outlining the purpose of the study and were shown images of the famous faces used in the subsequent experiment, which they were required to successfully name to ensure they are familiar with these. Participants read the ethical considerations and signed consent forms as approved by the Ethics committee of the University of East London.

#### Stimuli and procedure

**Figure 3:**
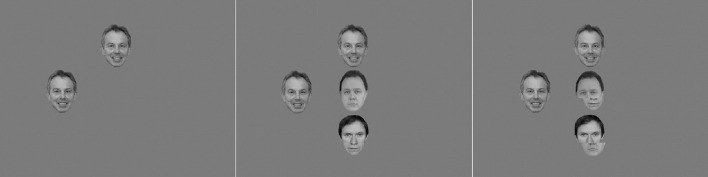
**Example of a trial display in the congruent condition with a relevant set size of one (left panel), three (middle panel) and with misaligned non-target faces (right panel) in Experiment 2**. Note: The versions of the faces shown here differ from the images used in the actual experiments due to copyright limitations. The image of Tony Blair is a cropped version of an originally larger photograph depicting Tony Blair and Robert M. Gates. As a work of the U.S. federal government, the image is in the public domain. The author holds the copyright to the other two images, and has permission of the persons to use them for publication.

Participants were positioned in front of a 15″ CRT monitor at a distance of approximately 60 cm. Each display comprised the target face at fixation or with its center 3 cm above or below fixation. In the low load condition, the target face was presented alone at either of these positions. In the high load condition and the misaligned condition the target face was also shown in one of these three positions, but two other anonymous faces (both as normal intact images in high load, or both misaligned in the misaligned condition) were presented as non-targets in the other two locations. Participants were required to indicate with a speeded key press (the “1” and the “2” key on the keyboard number section) whether the famous face was a politician or a film star. All faces depicted people of an apparent age between approximately 40 and 55 years, see Lavie et al. (2003). Examples of politicians are David Cameron or George Bush, and examples of film stars were George Clooney and Hugh Grant (as in Experiment 1). Four faces of famous politicians and four famous film stars were used (the same as in Experiment 1) and the allocation of face identities as a target per trial was randomized. The two non-famous male faces which served as non-targets (in the high load and misaligned conditons) were from a pool of twelve non-famous faces (these were the same images used as in Thoma and Lavie, 2013; Thoma, 2014). For the misaligned condition the non-famous faces shown as non-targets were manipulated versions of the original images of the anonymous faces, so that the top and bottom parts of the faces were separated (cut horizontally below the bridge of the nose and above the mouth section) and combined with the top and bottom parts of other faces resulting in amalgamations of two different faces (see Figure 3). The top and bottom halves of the non-famous faces were moved apart slightly vertically (degree of separation was 25%, see Figure 3). The aligned versions were the same face composites but aligned to form a whole face.

In addition to the target and non-targets in the center of the display, a peripheral distracter face was presented 4 cm either to the left or right of fixation. This face was either the same (congruent) as the target face, or from the opposite category (incongruent) (see Section Notes). The face images were presented as a grayscale image with a standardized vertical size of 3 cm (2.86 degrees of visual angle) for targets and non-targets and 3.4 cm (3.24 degrees) for distracters. Distracter faces were positioned with their center 4 cm (3.82 degrees) to the left or right of the center. E-prime 2 was used to run the experiment. The category and position of the target face relative the identity and position (left or right of the center) of the distracter face were counterbalanced across all trials. After a practice block, three blocks of 72 trials were presented, each displayed until the participant had responded or 3 s had elapsed. If participants made an identification error or did not respond within 3 s, they heard a beep tone.

### Results

**Figure 4:**
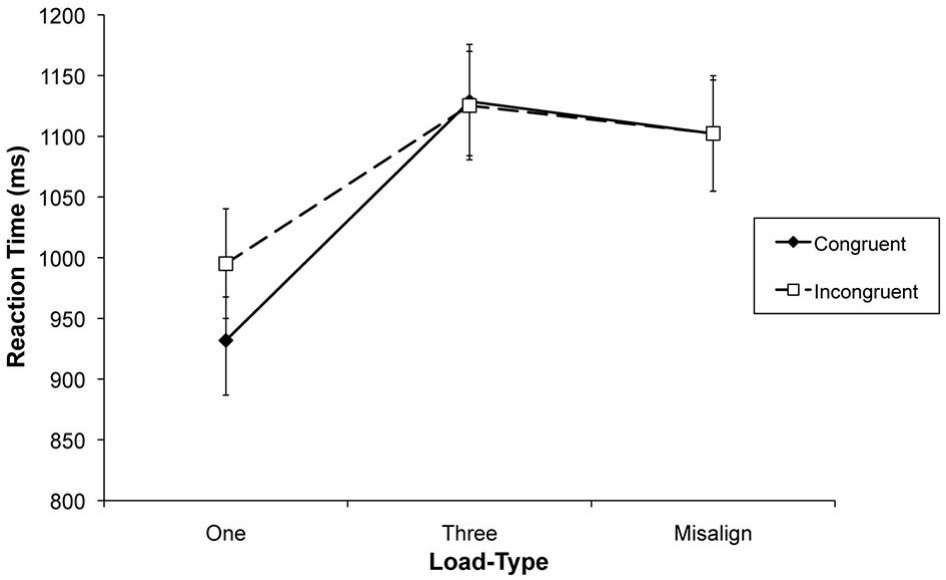
**Mean reaction times in the face classification task of Experiment 2 as a function of load-type and congruency**. Error bars represent standard error of the mean.

Trials with RTs shorter than 150 ms (0.3% of the trials) and incorrect responses were excluded from the analyses of RTs. Figure 4 displays the mean correct RTs as a function of the experimental factors. A repeated measures ANOVA was conducted with the independent variables of congruency (congruent and incongruent) and load-type (low load, high load, and misaligned). The assumption of sphericity for the factor load-type could not be upheld, therefore, we report Greenhouse-Geisser corrected results. There was a main effect of congruency, with congruent trials being responded to faster than incongruent ones, *F*_(1, 19)_ = 7.37, *p* = 0.014, partial η^2^ = 0.280. There was also a main effect of load-type, *F*_(1.53, 29.05)_ = 72.87, *p* < 0.001, partial η^2^ = 0.793. Planned comparisons showed that RTs in the misaligned trials were slower than in the low load condition, *F*(_1, 19)_ = 60.84, *p* < 0.001, partial η^2^ = 0.762, and the RTs in the high load conditions were slower than in the misaligned condition, *F*_(1, 19)_ = 5.32, *p* = 0.033, η^2^ = 0.219.

There was a congruency by load-type interaction, *F*_(1.88, 35.68)_ = 5.02, *p* = 0.013, partial η^2^ = 0.209. This was explained by a significant difference between congruent and incongruent trials under low load, *t*_(19)_ = 3.76, *p* < 0.01, but not in the other load conditions, both *t*s < 1. Error rates were not normally distributed and therefore analyzed with a Friedman ANOVA which showed no difference between conditions, $\chi_{\left(5\right)}^{2}$ = 4.76, *p* = 0.446 (overall *M* = 4%, *SD* = 1%; see Table 2).

**Table 2:** **Mean error rates (in percent) and Standard deviation for conditions in Experiment 2**.

	**Congruent**		**Incongruent**	
	***M***	***SD***	***M***	***SD***
Set size 1	0.05	0.06	0.03	0.03
Set size 3	0.04	0.04	0.03	0.03
Set 3 misaligned	0.05	0.05	0.04	0.04

Experiments 1 and 2 therefore replicated the findings of Thoma and Lavie (2013) and Thoma (2014), showing that face-processing seems to depend on capacity limits that are category-specific, but that do not rely on configural representations of faces. To further explore the locus of capacity-limited processing in regards to face-specificity, Experiment 3 uses a different manipulation of face images, polarity reversal.

## Experiment 3

Experiment 3 again examined face-specific capacity in a categorization task using famous faces as targets, and non-famous faces as non-targets in a visual search task, this time using polarity-reversed faces. As in Thoma (2014), the addition of non-target faces to the (central) search task should require face processing resources and eliminate interference from a distracter face. In addition, Thoma (2014) found face–specific capacity limitations even with inverted non-target faces, and Experiment 2 of the present study with misaligned versions of faces. In the current experiment, we studied the effect of adding non-target faces to the search set that were shown in a polarity-reversed (image negative) version of the original image. As mentioned above, previous research so far suggests that the capacity for face perception is only depleted by face images with intact (in terms of pigmentation and 3D information from shading) face-parts, therefore the addition of polarity-reversed faces should not affect congruency effects compared to the low load condition.

A further interest was in potential effects of practice on the congruency effect under different load conditions. If category-specific limits in face recognition are mediated by an encapsulated “face”-module (Fodor, 1983) then we would not expect any practice effects such that congruency effects are changed after repeated exposure to high load situations. In other words, we would expect that the congruency effect appears even in high load (2 intact faces as non-targets) after extensive training. To test this idea, we extended the number of trials and blocks as well as the number of participants (for increased power) in Experiment 3.

### Materials and methods

#### Participants

Thirty-five participants (21 female) were recruited on a voluntary, unpaid basis, among psychology students at the University of East London. The mean age of participants was 28.44 with ages ranging from 18 to 49. All reported normal or corrected-to normal vision. Participants read a document outlining the purpose of the study and were shown images of the famous faces used in the experiment and all successfully named them. Participants read the ethical considerations and consent forms as approved by the Ethics committee of the University of East London.

#### Stimuli and procedure

**Figure 5:**
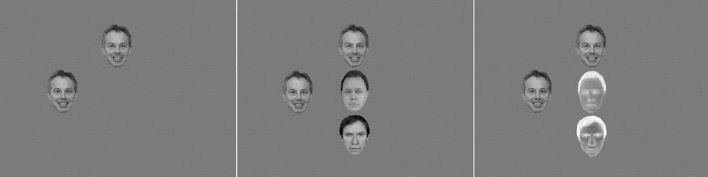
**Examples of a trial display in the congruent condition with relevant set sizes one (left panel), three (middle panel), and polarity-inversed non-targets (right panel) in Experiment 3**.

The design and set-up of the experiment was identical to Experiment 2, except for the following changes: Twenty-four male faces were presented which comprised of six famous politicians (adding Nicolas Sarkozy and Gordon Brown), six famous film stars (adding Brad Pitt and Michael Douglas), and twelve unfamiliar faces which served as non-targets in conditions with set size 3 (adding 2 either polarity reversed or 2 intact faces to the search display containing the target). The condition containing misaligned non-target faces in Experiment 2 was replaced with a “negative-high-load” condition: In the negative-high load condition, the target face was presented together with two polar-reversed non-target faces, which were image-manipulated versions of the 12 anonymous faces used in the high load (see Figure 5). There were 8 blocks of 72 trial screens (576 in total), after an initial practice block. The identity and position of the target face, the identity and position of the distracter face were counterbalanced across all trials.

### Results

Trials with response times below 150 ms were excluded (0.3% of all trials), and for the RT analysis only correct responses times were analyzed. A 2 (congruency) × 3 (load-type) × 8 (block) within-subjects ANOVA was performed on response times and error rates. Data from one participant were removed from the analysis because of high error rates (overall mean 25%).

**Figure 6:**
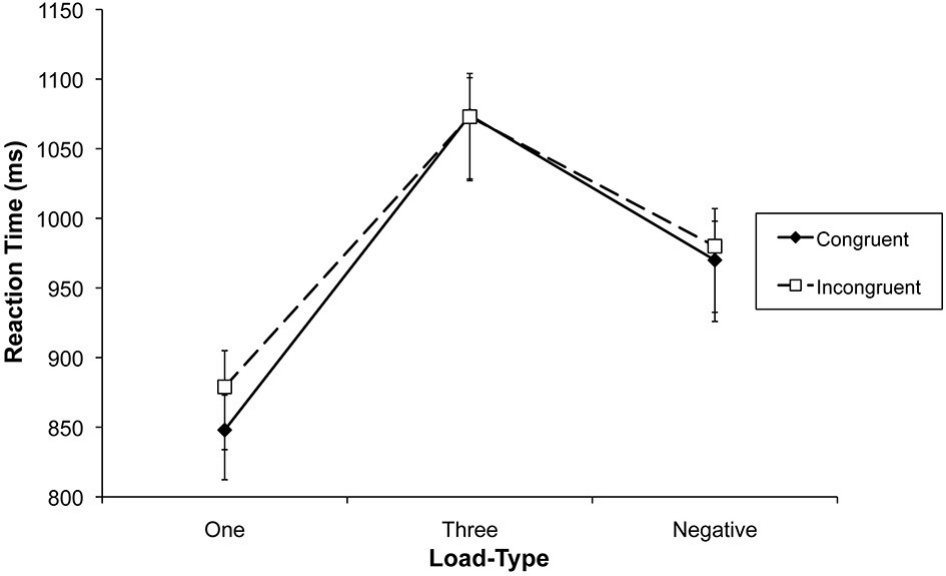
**Mean reaction times in the face classification task of Experiment 3 as a function of load-type and congruency**. Error bars represent standard error of the mean.

There was a significant effect of load-type, *F*_(2, 66)_ = 292.0, *p* ≤ 0.001, partial η^2^ = 0.889, with negative-face load trials slower than low load, *p* < 0.001, and high load conditions slower than negative-face load, *p* < 0.001. There was a main effect of congruency, *F*_(1, 33)_ = 9.34, *p* < 0.01, partial η^2^ = 0.221, with congruent trials being responded to faster than incongruent ones (see Figure 6). There was also a main effect (Greenhouse-Geisser corrected degrees of freedom) of block, F (3.36, 111.11) = 6.81, *p* = < 0.001, partial η^2^ = 0.171, with mean response time decreasing from Block 1 (*M* = 1017, *SD* = 176) to Block 8 (*M* = 939, *SD* = 178), demonstrating a significant linear trend, F(1, 33) = 15.40, *p* < 0.001, partial η^2^ = 0.318. There was a significant interaction effect between load-type and congruency, *F*_(2, 66)_ = 3.80, *p* = 0.027, partial η^2^ = 0.103, but there were no other significant interaction effects, all *F*s < 1.34. The interaction was explained by a significant difference in the congruency effect between low load and high load conditions, *F*_(1, 33)_ = 7.36, *p* = 0.01, partial η^2^ = 0.18. There was no significant difference in the congruency effect between high load and reversed-polarity conditions, *F*_(1, 33)_ < 1. Follow up *t*-tests showed congruency differences only under low, *t*_(33)_ = 3.87, *p* < 0.001, but not in the high load, *t*_(33)_ < 1, or reverse load condition, *t*_(33)_ = 1.28, *p* = 0.21. An equivalent error analysis showed no effects, all *F*s < 1.33, see Table 3.

**Table 3:** **Mean error rates (in percent) and Standard deviation for conditions in Experiment 3**.

	**Congruent**		**Incongruent**	
	***M***	***SD***	***M***	***SD***
Set size 1	0.04	0.07	0.04	0.06
Set size 3	0.04	0.06	0.04	0.07
Set 3 reversed	0.04	0.06	0.04	0.07

To summarize, as in Experiment 2, the data show congruency effects from the peripheral distracter face in the low load condition, which had only a single target face as competition, whilst addition of two normal-polarity anonymous faces was sufficient to eliminate the distracter interference effect. Addition of two polar-reversed faces also eliminated the observed difference in mean RT. These effects did not change over time with practice.

**Figure 7:**
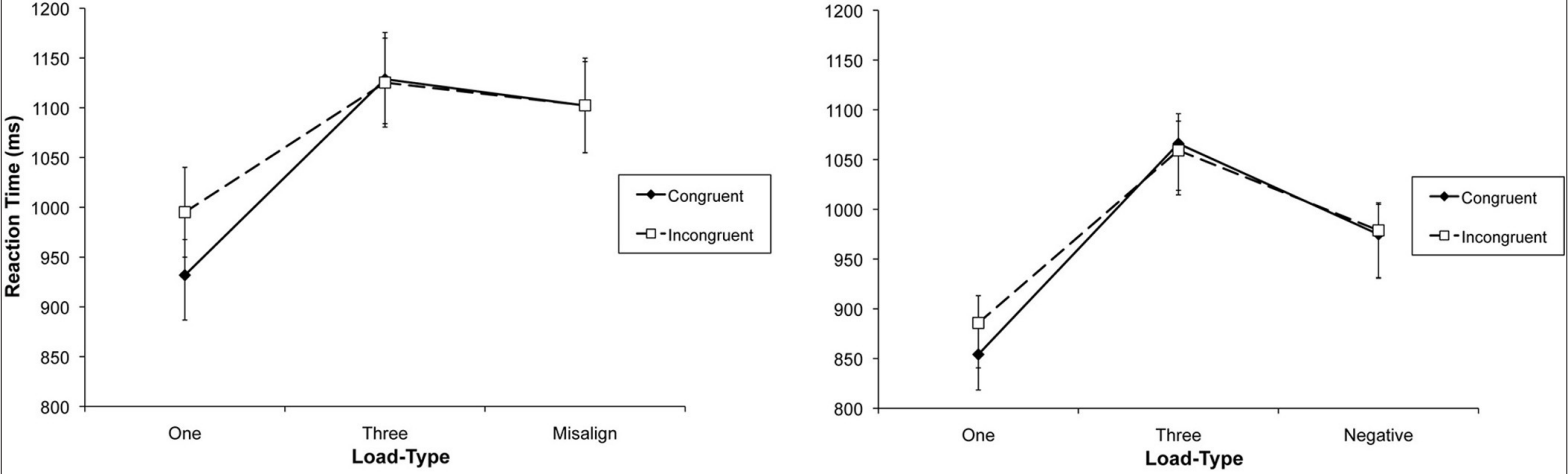
**Mean response times as a function of load-type and congruency in Experiment 2 (left panel) and Experiment 3 (right panel)**. The combined analysis was based on the same face identities in Experiments 2 and 3, which meant that for Experiment 3 only trials were included with the same eight target face identities as in Experiment 2.

In a final analysis, we compared the load effects between Experiment 2 and 3. First, we reduced the data set of Experiment 3 and included only trials containing the same famous faces (four politicians and four film stars) as used in Experiment 2 (see Figure 7). Then we ran a split-plot ANOVA with the combined results of the two experiments (as the between subjects factor). There were the usual effects of congruency, *F*_(1, 52)_ = 9.73.0, *p* < 0.01, partial η^2^ = 0.16, and load-type, *F*_(2, 104)_ = 227.42, *p* < 0.001, partial η^2^ = 0.81, and interaction between these two, *F*_(2, 104)_ = 10.35, *p* < 0.001, partial η^2^ = 0.17. There was a marginal main effect of experiment, *F*_(1, 52)_ = 3.96, *p* = 0.052, partial η^2^ = 0.07, reflecting somewhat longer response times in Experiment 2. There was an interaction between experiment and load-type, *F*(_2, 104)_ = 6.40, *p* < 0.01, partial η^2^ = 0.11: While there was no significant difference in the search slopes of Experiment 3 and Experiment 2 between low load and high load, *F*_(1, 52)_ = 2.78, *p* = 0.10, partial η^2^ = 0.05, the differential manipulation of non-target faces had a significantly stronger effect on target search slopes between high load and face manipulation (polarity-reversed vs. misaligned), *F*_(1, 52)_ = 10.35, *p* < 0.001, partial η^2^ = 0.17. Polarity-reversed faces slowed the search task significantly less than misaligned faces, which in turn had similar search slopes to normal non-target faces. Importantly, there were no other interaction effects, *F*s < 1.21, hence no differential impact from the type of experiment on congruency effects.

## Discussion

The investigation reported here provides further evidence that processing of distracter faces is dependent on search displays that are face-specific. In addition, the experiments described here tested systematically whether this face-specificity can be explained by so-called “configural” properties of presumed face representations. Experiment 1 confirmed previous findings (Lavie et al., 2003) that increasing perceptual load in a semantic, word-based search task has no effect on the automatic processing of a peripheral distracter face, at least to a load level of six letter strings. In Experiment 2, when the central search task included only a single target famous face, a lower mean RT was observed for the congruent compared to the incongruent condition, suggesting that the distracter face was processed automatically. The addition of two non-famous faces to the search task removed interference from the distracter face, consistent with the finding that face-specific processing capacity is being exhausted when more than two faces (target and distracter) were present in the display (Thoma and Lavie, 2013), and therefore little or no spare resources would be automatically allocated to peripheral stimuli. Importantly, when two misaligned (Experiment 2) or polarity-reversed faces (Experiment 3) were added to the search task as non-targets, interference from distracter faces was again eliminated, suggesting that these stimuli had exhausted the available face processing capacity.

The present data thus counter previous research suggesting that the processing of faces has no capacity limits (Lavie et al., 2003; Neumann et al., 2011). The findings do confirm and extend the observations that faces exhibit the properties of a dedicated processing module which functions largely automatically and separately from processing of non-face stimuli such as letter strings (Thoma and Lavie, 2013; Thoma, 2014) and common objects (Lavie et al., 2003, 2009). The former studies have shown that when participants were asked to perform a visual search task looking for a famous face target in the center adding anonymous faces eliminated distracter interference from a congruent or incongruent face flanker. Surprisingly, Thoma (2014) found that this result holds even when non-targets were presented upside down, indicating that face-specific capacity limits were not mediated by holistic properties of faces (in the sense of preserved first-order relations between parts). One of the main questions of the current paper was therefore whether other manipulations related to configural processing would help to determine the nature of face properties underlying the observed capacity limits.

The manipulation used in Experiment 2 of employing misaligned face parts as non-target stimuli showed that capacity limits are not mediated by so-called second-order properties of faces (i.e., the distance between face parts). This manipulation leaves first-order relations (eyes above nose, nose above mouth) intact, but disrupts typical second-order relations (e.g., that the distance between parts). This confirmed and extended Thoma's (2014) finding that the hallmark of face processing—holistic configuration—seems not to play an important role in determining capacity limits. Thus, the basis for face-processing limitations may lie in processing of face parts rather than their relations to each other. In Experiment 3 therefore we used polarity-reversed faces as non-target load inducing stimuli, because polarity-reversal was reported to severely reduce processing of face parts (e.g., Kemp et al., 1990) or surface pigmentation (Liu et al., 2000), yet leaves the holistic configuration of face-parts intact. Surprisingly, and against our hypothesis, polarity reversed non-target faces still diminished interference effects from distracters, and this effect could not be explained by practice. Thus, it is still unclear what constitutes the exact nature of capacity limits. Nevertheless, our findings help to narrow down the representational locus of category-capacity limits for faces. This is because we already know from previous work about at least one type of face-image manipulation that does not affect congruency effects.

In one of Thoma and Lavie's (2013) experiments non-target faces in the search set were replaced with phase-scrambled faces: the original image versions of anonymous non-target faces were submitted to a 2-D Fast Fourier transformation, which randomizes the phase spectrum, while keeping the amplitude (power spectrum) of the image intact (McCarthy et al., 1997; Jenkins et al., 2003). In addition, the outline of the scrambled “faces” was similar to those of the intact faces (e.g., with a discernable chin area). Although the phase-scrambled version had a similar outer shape and the same physical energies as the originals, adding these faces to the search did not reduce the congruency effect, unlike the original non-target faces. Hence, our conclusion is that capacity limits for face perception are determined by visual features that reflect basic visual face parts, though these need not be detailed and specific enough to allow face identification, nor need they be arranged in specific face-like configurations. It is worth noting that all three manipulations of faces tested so far in face-load studies—inversion, misalignment, and polarity-reversals—allow the immediate categorization of the stimuli as faces (e.g., see Itier et al., 2006; Laguesse and Rossion, 2013), while they are reported to significantly impair identification (or subordinate-level recognition).

There is a potential alternative to our proposal that distracter processing depends on perceptual load, which is that our data may be explained by a so-called “dilution” account of distracter processing. While perceptual load postulates a limited resource for processing targets, non-targets, and distracters to explain reduced target-distracter interference effects, dilution accounts (Tsal and Benoni, 2010; Wilson et al., 2011) attempt to explain reduced distracter processing effects by arguing that adding more items to a search display is “diluting” the processing for all stimuli (non-targets and distracters) in the response competition paradigm due to some form of crosstalk among stimulus features. Thus, according to these accounts assuming featural crosstalk, any additional item in the display should dilute distracter processing. The current observations of Experiments 2 and 3 of diminished distracter processing in all high load conditions could therefore be interpreted as the result of simply adding any stimulus, which would diminish distracter processing. However, we already know from Thoma and Lavie's study (Thoma and Lavie, 2013; Experiment 4) that this is not the case, as adding phase-scrambled non-target versions of faces did not diminish (or “dilute”) distracter face processing. Furthermore, the fact that polar-reversed non-target faces eliminate distracter processing just as much as misaligned faces while the latter result in steeper search slopes is noteworthy. It may indicate that while intact face parts (misaligned non-targets) are more important than feature relations for search performance (polarity-reflected non-targets), both affect the processing of multiple face perception. But more importantly, as increased search slopes indicate increased similarity between target and non-targets (Duncan and Humphreys, 1989, 1992), we can conclude that mere similarity between different non-target faces (scrambled, misaligned, and polarity-reversed) and a target face cannot explain modulation of distracter processing, as would be predicted by a dilution account. Finally, work on dilution accounts (using letters as stimuli) has argued that knowing the color of a target-distracter combination should eliminate distracter interference (dilution) effects, as such a grouping would make it easier for the observer to exclude the different colored non-targets (Chen and Cave, 2013) from processing and therefore causes no dilution effect (i.e., imposes no load). In terms of dilution, we would therefore have expected similar results for polarity-reversed conditions, namely that polarity-reversed non-targets would not dilute distractor processing to the same extent as intact non-target faces would. Instead, we found equal reductions in distractor processing for intact and polarity-reversed non-targets. Thus, although the current experiments were not designed to test between “dilution” accounts and “perceptual load” accounts, it seems the latter one is the most parsimonious explanation given the present data (see also Lavie et al., 2009; Thoma and Lavie, 2013).

The results from Experiment 2 (and Experiment 3) are similar to the findings of Thoma (2014), and implies that the capacity bottleneck for faces occurs before configural (here second-order relational) processing. However, since these aspects of processing are central to the special status of faces, the question arises how the observations could account for face-specific capacity limitations (Thoma and Lavie, 2013). One possibility is that face-processing limits are determined by the processing of specific features of the face and there is research that suggests a featural route to face recognition. For example, Schwaninger et al. (2009) found that part-scrambled faces were not more difficult to recognize than the faces in which features were placed in their first-order relational positions but with distorted metrical distances. Gilad et al. (2009) hypothesized that the poor recognisability of negated faces might be due to disruption of stable polarity relations around specific facial features. Using a series of “contrast chimeras” (faces shown in negative, apart from features such as the eyes and mouth), they demonstrated that ordinal relationships around the eye area were major determinants of recognisability. A number of other researchers have identified the region around the eyes as particularly important in recognition of faces under normal lighting conditions (Gilad et al., 2009; Sormaz et al., 2013) and it has been shown that this is true regardless of face orientation (Sekuler et al., 2004) or how long subjects have been practicing (Gold et al., 2004). Gaspar et al. (2008) suggested that the reason upright, normal polarity faces are more easily recognized than inverted or polar reversed faces is that extensive practice results in a more efficient strategy for sampling information (in particular the regions around the eyes), therefore benefitting normal upright faces. In general, a substantial body of behavioral work now suggests a special status for eye/eyebrow features as being of primary importance for face recognition, followed by mouth features (Sekuler et al., 2004; Caldara et al., 2010; Gold et al., 2012).

In conclusion, we present further evidence for category-specific processing limitations in face recognition. Peripheral distracter faces are perceived under low and high load during a central visual search task, unless the central search comprises of faces. This study shows that these capacity limits are not constrained by metric configurations of face parts, nor do they rely on strictly veridical face parts alone. Future research will have to further probe the exact nature of representations underlying face-specific attentional resource limitations.

## Notes

In line with previous studies (e.g., Lavie et al., 2003), the target and the distractor were identical in the congruent condition, which meant they matched in terms of both visual characteristics and identity. In order to differentiate between effects driven by target-distractor congruency in terms of visual stimulus characteristics vs. stimulus identity, one could present different target and distractor images (but from the same category) in the congruent condition. However, previous work has demonstrated that compatibility effects may be hard to interpret in such cases, as the compatible condition now consists of non-matching stimulus pairs (e.g., Santee and Egeth, 1982). Further work is needed to examine this issue.

## Author contributions

VT initiated the work, designed the studies, analyzed the data, and worked on the write-up; NW designed and conducted the studies, analyzed the data, and worked on the write-up; JD was involved in discussions setting up the studies, interpreting the results, and worked on the write-up.

### Conflict of interest statement

The authors declare that the research was conducted in the absence of any commercial or financial relationships that could be construed as a potential conflict of interest.

## Footnotes

^1^There is a debate about the exact definition of the terms holistic, configural, and feature or part-based processing (e.g., see Maurer et al., 2002; Peterson et al., 2003; Laguesse and Rossion, 2013; Richler and Gauthier, 2014). As a general framework, holistic processing suggests the perceptual integration of information of face parts, representing detailed spatial relational (e.g., nose-mouth distance) or metric (e.g., distance of nose and eye edges) information. Part-based processing means local processing of individual features, based on contour boundaries in the face.
